# Finite Element Analysis of Tunable Composite Tubes Reinforced with Auxetic Structures

**DOI:** 10.3390/ma10121359

**Published:** 2017-11-27

**Authors:** Hubert Jopek

**Affiliations:** Institute of Applied Mechanics, Poznan University of Technology, ul. Jana Pawła II 24, 60-965 Poznan, Poland; hubert.jopek@put.poznan.pl

**Keywords:** tunable, auxetic, composite, tube, stent, thermoauxetic, magnetorheological

## Abstract

A tubular composite structure that is built of two materials, characterized by different Young moduli, is analysed in this paper. The Young’s modulus of one of these materials can be controlled by external conditions e.g., magnetic or electric field, temperature etc. The geometry of the reinforcement is based on typical auxetic re-entrant honeycomb cellular structure. The influence of this external factor on the behaviour of the stretched tube is analysed in this paper. Also, the possibility of creating a tubular composite structure whose cross-section is either shrinking or expanding, while stretching the tube is presented.

## 1. Introduction

Auxetics have become more and more popular over the recent years. Not only are they extensively investigated by researchers, but also auxetics or auxetic-like structures are already used commercially. It has been shown that auxetic behaviour i.e., negative Poisson’s ratio results from the specific geometry of material structures. Some of the first analyses of re-entrant honeycomb structures were presented by Kolpakov [1] and Gibson [2]. Nowadays, many geometrical patterns are known to be responsible for auxetic behavior [3]. Such behaviour on the molecular level was first presented by Wojciechowski [4,5] and Evans [6], and also by Kang et al. [7] and Grima for single molecules [8], as well as by Goldstein for crystals [9,10]. Macroscopic auxetic structures can be divided into several classes: foams [11] and porous structures [12,13], re-entrant structures [14,15], chiral structures [16], non-chiral structures [17,18] rotating rigid or semi-rigid units [19], and many others.

Auxetic structures can also result from specific material distribution in composite materials, even though all of the constituents are characterized by positive values of the Poisson’s ratio, an analysis of such structures was presented by Strek and Jopek [20,21]. Czarnecki et al. [22,23] presented some results concerning composites built by optimal distribution of Young’s modulus within elastic and isotropic by means of topological optimization. Auxetic behaviour as a result of hard inclusions in the soft matrix was shown by Pozniak [24] and Kochman [25]. Another class of auxetic composites are multiphase structures, in which at least one constituent exhibit the negative value of the Poisson’s ratio. The influence of auxetic fibres reinforcement on the mechanical behaviour of a composite under bending load was studied by Jopek [26]. Bilski and Wojciechowski [27] investigated the possibility of changing the value of effective Poisson’s ratio in layered composites with an auxetic layer built of hexamers. The torsional behaviour of composite beams was studied by Strek [28,29].

A special group of materials contains so-called smart materials i.e., materials with controllable properties. In recent years, several articles have been published on using auxetics in smart materials. Ren et al. showed that the auxetic performance can be tuned by the geometry of microstructures, while the strength and stiffness can be tuned by the plasticity of the base material, while maintaining the auxetic performance [30]. Jopek and Strek [21] analysed thermoauxetic composite in which the value of the effective Poisson’s ratio was tunable and switched from positive to negative value according to the temperature applied, although all of the constituents are characterized by positive values of the Poisson’s ratio. Similar behaviour was demonstrated later in the case of bi-material unit cells by Li, Dong and Lakes [31,32]. Mechanical properties of materials could also be controlled by the electric or magnetic field applied. Magneto- and electroelastic properties of materials have been analysed by many researchers [33,34,35] In particular, there are polymer materials whose elastic properties are strongly dependant on external magnetic field. Such materials are called magnetorheological elastomers, and their properties has been studied by Kukla et al. [36] and Varga et al. [37]. Alderson et al. [38] discussed piezomorphic materials i.e., materials that change smoothly their shape in response to mechanical stress. Piezoelectric bimorph composite with auxetic phase used for increased power output in vibration energy harvesting [39]. The influence of magnetic field and the behaviour of magneto-auxetic systems have been investigated by Grima et al. [40] and Dudek et al. [41,42]. A very comprehensive research has been presented by Danas [43], who described both theoretically and experimentally the behaviour of magnetoelastic materials with auxetic microstructure. Raghunath [44] also studied magnetoelastic properties of Galfenol, which exhibit auxetic behaviour. 

Tubular structures are one of the most common mechanical elements used in engineering. Although the cross-sectional shape of the tube can be in fact arbitrary, it is a very common to use the simplest possible, i.e., circular, shape. Such circular hollow cross-sections have been found in natural structures e.g., body parts like trachea or blood vessels, stalks many plants etc. When it comes to engineering, using tubular structures has just become a necessity in the process of structural design. Apart from an obvious application as parts of pipelines, tubular elements are also used in construction as they exhibit good mechanical performance when compared to solid cross-sectional elements with the same material consumption. It is possible to build tubular elements, e.g., stents with use of auxetic materials and structures [45]. Duc et al. [46] analysed the nonlinear dynamic response and vibration of sandwich auxetic composite cylindrical panels built of three layers, in which the top and bottom outer skins are isotropic aluminum materials, while the middle core layer is auxetic. Several articles have already been devoted to the mechanical properties and behaviour of auxetic tubes. Ren et al. investigated the behavior of auxetic tubes that were made of different materials [47]. Goldstein et al. [48] analysed mechanical properties of two-layer tube composites build of auxetic and nonauxetic material. Zhang et al. analysed the possibility of manufacturing auxetic materials with use of tubes and corrugated sheets [49]. Scarpa et al. [50], and Yao et al. [51] presented the analyses of Single-Walled Nano Tubes. The method in transforming conventional silicone rubber composites to auxetic robust rubbers in the case of graphene and carbon nanotube was published by Valentini et al. [52]. The influence of auxetic phase in concentric composite has been analysed by Strek and Jopek [53], whereas the structure and properties of auxetic oesophageal stents have been studied by Ali et al. [54]. The mechanical behavior of auxetic tubes, and the study of the influence of selected geometrical parameters was presented by Karnessis et al. [55]. The method of manufacturing auxetic stents was proposed by Bhullar et al. [56]. Theoretical and experimental research concerning tubes that were filled with auxetic foams were discussed by Mohsenizadeh et al. [57].

Recently, auxetic materials have been extensively studied due to their enhanced mechanical properties, e.g., enhanced stiffness of composites reinforced with auxetic structures. The possibility of using these structures in composites of controllable mechanical properties, so-called smart materials, is very promising and concerns the research in the field of the latest research in materials mechanics. In this article, an analysis of the behaviour of composite tube reinforced with auxetic phase is analysed. The composite is built of two constituents. It is assumed that the Young’s modulus of one of these constituents is dependent on an external factor, such as magnetic field, electric field, or temperature, whilst the other stays unaffected. The aim of the analysis is to present tubular composites based on auxetic structures, whose diameter could either shrink or expand in a controlled way depending on the external factor. Such a tunable tubular structures could be then analyzed in the application of fluid and bio-fluid mechanics, as well as the structural morphing element.

## 2. Composite Structure of the Tube

**Figure 1:**
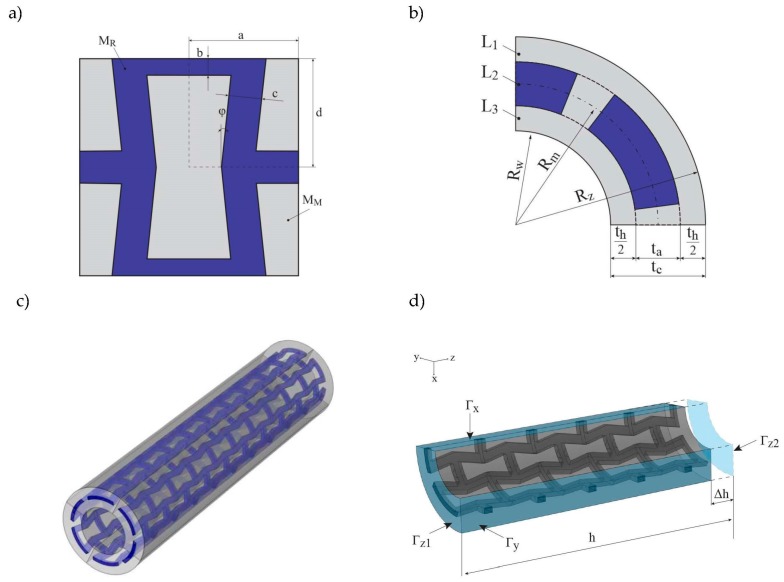
The geometry of composite tube: (**a**) composite unit cell of reinforcement layer, (**b**) quarter of cross-section with all of the layers marked (**c**) three-dimensional view of the whole tube, (**d**) the considered one-eighth of the tube with boundary conditions marked.

The reinforcement of the tube is built of auxetic unit cells based on typical re-entrant honeycomb geometry (see Figure 1a). The material of auxetic structure being the reinforcement is denoted as M_R_ (blue colour). The voids within the auxetic structure are completely filled with the matrix material M_M_ (grey colour), so that we obtain a solid bi-material composite layer (L_2_ see Figure 1b)*.* The thickness of the reinforcement L_2_ layer is denoted as *t_a_*, but the reinforcement layer can also be embedded in the M_M_ material so that the matrix creates also inner (L_3_) and outer (L_1_) layers, which isolate the reinforcement from the surroundings. Both od these layers are equally thick and their cumulative thickness is denoted as *t_h_*. Overall thickness of the tube is defined as *t_c_* = *t_a_* + *t_h_* The curvature of the circular cross-section of the tube is defined by the centre line that divides the thickness of the tube’s wall into halves. The radius of this centre line equals *R_m_*. Therefore, the inner (*R_w_*) and outer (*R_z_*) radii are defined as follows: *R_w_ = R_m_ − (t_a_ + t_h_)/2, R_z_ = R_m_ + (t_a_ + t_h_)/2* (see Figure 1b). The geometry of three-dimensional (3D) composite tube is presented in (Figure 1c). It is assumed that the even number *N_c_* of unit cells is distributed circumferentially, as well as the even number *N_z_* is distributed along the tubes height. Hence, the geometry is symmetrical with respect to the three main planes and only one-eighth part of the tube needs to be analysed. In order to determine the volumetric fraction of each constituent, one can make use of the symmetry of the unit cell and calculate only the quarter of the unit cell whose area is defined as *A = ad*. The area of the reinforcement can be expressed as follows:

(1) $$A_{R}=\left(d-b\right)\left(\frac{c}{\cos\left(\varphi \right)}+b\;\tan\left(\varphi \right)\right)+ab$$

and the area of the matrix:

(2) $$A_{M}=A-A_{R}$$

So, the share of each constituent in the layer of reinforcement (L_2_):

(3) SA,R=ARA,

(4) SA,M=AMA,

However, one needs to take into account the thickness of the tube’s wall and the fact that the reinforcement could be thinner due to matrix material that surrounds the reinforcement from the inner and outer side. The matrix material that forms the inner and outer layers could isolate the reinforcement that embedded in the tube, but it also diminishes its influence as the share of M_R_ material in the volume of the whole composite decreases. In that case, the wall could be split into three layers: internal layer L_3_ built of the matrix material only of volume *V*_1_, the middle layer L_2_ being the mixture of matrix material, and the reinforcement of volume *V*_2_*,* and finally the outer layer L_1_, also built only of matrix material only of volume *V*_3_ so the volume of each constituent is defined by the following formulae:

(5) $$V_{R}=S_{A,R}\times V_{2},$$

(6) $$V_{M}=V-V_{R},$$

where $V=V_{1}+V_{2}+V_{3}$ is the total volume of the tube. Hence volumetric share of each constituents is expressed as:

(7) SV,R=VRV,

(8) SV,M=VMV,

Smart materials whose properties are tunable via external conditions, such as electric or magnetic field, temperature etc. allow for creating more sophisticated structures, which could be also controlled by the same factor. In the case of the considered composite tube, it is built of two materials M_R_ and M_M_, and the material of the reinforcement is such smart material and its Young’s modulus that can be controlled by external factor, while the change of the Young’s modulus of the other material is negligibly small. In such a case, it is possible to investigate just the ratio of each constituents Young’s modulus instead of the values of each of them.

The reinforcement material M_R_ is characterized by Young Modulus *E*_R_ and the value of the Poisson’s ratio *ν_R_*, whereas the matrix material is characterized by *E_M_* and *ν_M_*. As it was shown by Milton [58], in the case of composites built of auxetic structure with all of the voids filled, that it is possible to preserve the negative Poisson’s ratio of this structure if the structure is stiff and the other material compressible, which means that the ratio of Young’s moduli is different from 1. Similar results have been presented by Goldstein [48]. Hence, the value of the effective Poisson’s ratio is strongly dependent on the values of Young moduli of constituents. When considering the fact that the key factor in the analysis is the ratio of both materials, Young’s moduli, it was assumed that *E_M_* = 1 and the value of *E_R_* varies in the following range *E_R_* = (0.001, 1000), which implies that the ratio *E_R_/E_M_* also changes in the same range. Moreover, it follows that simulation also cover cases in which the Young modulus of the reinforcement is constant and the Young’s modulus of the matrix is influenced by external factors. Either way, the ratio of Young’s moduli can be tuned. Although the effect of an external field on the Young’s modulus of magneto- and electro-elastic materials is usually not that great, it is possible to obtain the change of the Young’s modulus by two or three orders of magnitude with the change of temperature, hence, such wide range of analysis seems justified.

In case of the experimental investigation, magnetorheological elastomers could be used. These materials are built of a polymeric matrix with magnetic micro- or nanoparticles embedded. The structure that is considered could by obtained if the particles were distributed so that they create a re-entrant auxetic pattern. Another possibility is to use two polymers that can be bonded together, assuming that the Young modulus of one of the materials changes significantly with the change of temperature, while the other is almost constant in the same range of temperature e.g., two types of silicone rubber.

For simulation, it was assumed the basic values of *ν_M_* and *ν_R_* were assumed as 0.3, although the simulation for several different values of *ν_R_* was performed in order to investigate its impact as well. Symmetry boundary conditions are used on *Γ_x_*, *Γ_y_,* and *Γ_z_*_1_ boundaries and prescribed displacement along the z-axis is assumed on the *Γ_z_*_2_ see Figure 1d, the prescribed displacement ∆h=0.1h.

The change of *E_R_* results in the change of effective Poisson’s ratio of the composite, which in the case of flat geometry would by described as $\nu =-\frac{\varepsilon_{t}}{\varepsilon_{l}}$, where $\varepsilon_{t}$—transverse strain, $\varepsilon_{l}$—longitudinal strain along the stretching/compression direction. However, when considering the tube, the effective Poisson’s ratio of stretched (compressed) tube is calculated on the basis of the change of the length of its circumference, which could be expressed simply by its diameter or radius. Hence, the effective Poisson’s ratio of the axially stretched tube is given by the following [39]:

(9) $$\nu_{eff}=-\frac{\bar{\varepsilon_{c}}}{\bar{\varepsilon_{z}}}$$

where $\bar{\varepsilon_{c}}$—average circumferential strains and $\bar{\varepsilon_{c}}=\frac{∆R}{R}$, and $\varepsilon_{z}=\frac{∆h}{h}$. Given the fact that the composite material properties are not homogeneous in the cross-sectional area, one should expect that the deformed cross-section could diverge from its initial circular shape. Hence, both longitudinal and circumferential strains are averaged. 

## 3. Results

Numerical analysis was performed with the use of Comsol Multiphysics FEM software. Linear elastic materials are assumed for both of the materials. The mesh was built of about 20,000 tetrahedral elements. Geometrical parameters of the tube: *R_m_* = 1, *N_c_* = 6, *N_z_* = 10, *t_a_* = 0.1, *t_h_* = {0, 0.2}. The aim of the analysis is to present the influence of the change of reinforcement elastic modulus on the behaviour of the tube.

### 3.1. Case 1

**Figure 2:**
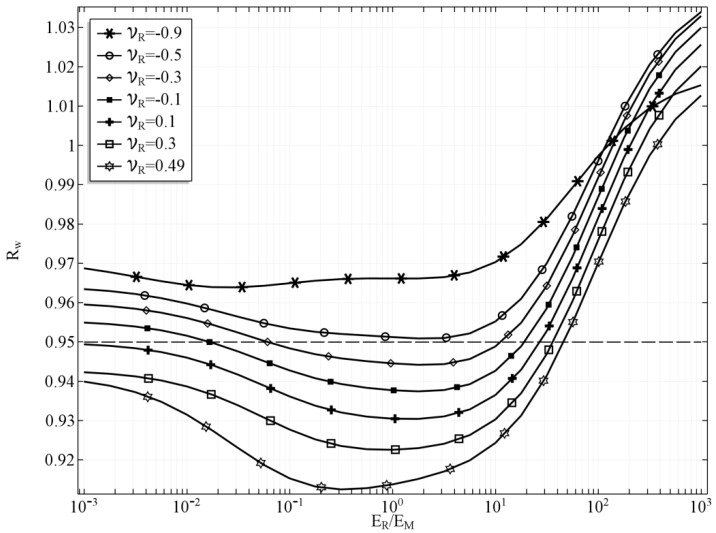
The inner radius of the tube *R_w_* with respect to *E_R_/E_M_* ratio for selected values of *ν_R_*, *t_h_* = 0.

In the first case, the results were obtained for the case, in which *t_h_* = 0, which means that the tube’s wall is of the same thickness as the reinforcement layer so no additional inner and outer layer occur. The volumetric share of the phase of the reinforcement $S_{V,R}\;$= 0.43. The change of the inner radius *R_w_* of the stretched tube with respect to the Young’s moduli ratio is presented in the Figure 2. Several values of the Poisson’s ratio of the reinforcement material are presented in order to investigate the influence of this parameter, as well while the value of the Poisson’s ratio of the matrix is kept constant *ν_M_* = *0.3.* The dashed line in the plot represents the inner radius of the tube before deformation, just for the reference. If the ratio *E_R_/E_M_* = 1 and *ν_R_ = ν_M_,* then the tube is built of isotropic homogenous material and it behaves as such.

One can observe that as the *E_R_/E_M_* diverge from 1 the radius of the tube also starts changing. However, the influence of the ratio *E_R_/E_M_* is far more significant if the ratio is greater than 1, which means that the material of the reinforcement is stiffer. This effect results from the fact that the reinforcement material creates auxetic structures, and the stiffer the reinforcement, the stronger it influences the behaviour of the whole tube. It is clearly noticeable that it is possible to obtain a tube that would either shrink or expand depending on the external factor that would change the reinforcement Young’s modulus. Moreover, such change is possible even for a relatively small change of this Young’s modulus, If *E_R_* is greater than *E_M_* by about one order of magnitude, then we can obtain a tube that would change its radius from 0.93 up to 0.97, which means that the cross-section of the tube would change by almost by 10%. Furthermore, a very interesting result is obtained in the case in which the ratio is changed to its reciprocal so that the matrix is stiffer than the auxetic reinforcement. The tube behaves similarly to the one with stiffer reinforcement, although the effect of changing the Young’s moduli ratio is smaller. 

The value of the Poisson’s ratio of the reinforcing material was also studied, and, naturally, it also influences the behaviour of the whole composite. The impact of changing the value of *ν_R_* decreases as the ratio *E_R_/E_M_* diverge from 1. For a completely isotropic material of the tube, the diameter of the stretched tube is minimal, however, this relation is dependent on the Poisson’s ratio. In the case of *ν_R_* = 0.49, the minimum value of the radius is obtained if the value of the ratio *E_R_/E_M_* < 1. For all the values of *ν_R_* < 0.3 the minimum radius of the deformed tube is obtained if *E_R_/E_M_* > 1. It is clearly noticeable that the value of *ν_R_* is also significant, and it enhances the auxetic behaviour of the reinforcement structure as *ν_R_* tends to −1. In consequence, lower values of *ν_R_* diminish the effect of the changing *E_R_/E_M_* ratio. For clarity reasons it was assumed that the external factor influences only the *E_R_/E_M_* ratio. So, in each case of *ν_R_*, the *E_R_/E_M_* ratio was the only control variable. It is important, however, to remember that the Poisson’s ratio can be also dependant on such phenomena as a magnetic field, electric field, or temperature. Hence, the radius of the tube could change in a wider range if both Young’s modulus ratio and the values of the Poisson’s ratio were controlled simultaneously by this external factor.

**Figure 3:**
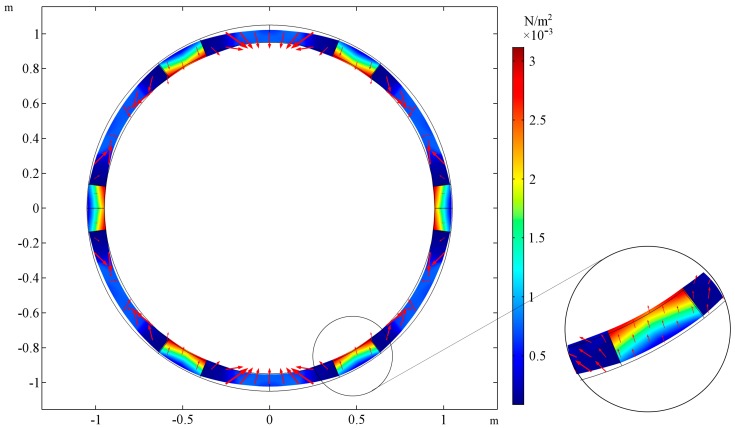
The deformation of the cross-section of the stretched tube and stress field for *E_R_/E_M_* = 0.001, the auxeticity of the reinforcement’s structure is dominated by the behavior of the matrix material. The tube’s wall thickness is equal to the reinforcement thickness, inner and outer layers (L_1_, L_3_) do not occur: *t_h_* = 0. The area of stress concentration is magnified on the right.

**Figure 4:**
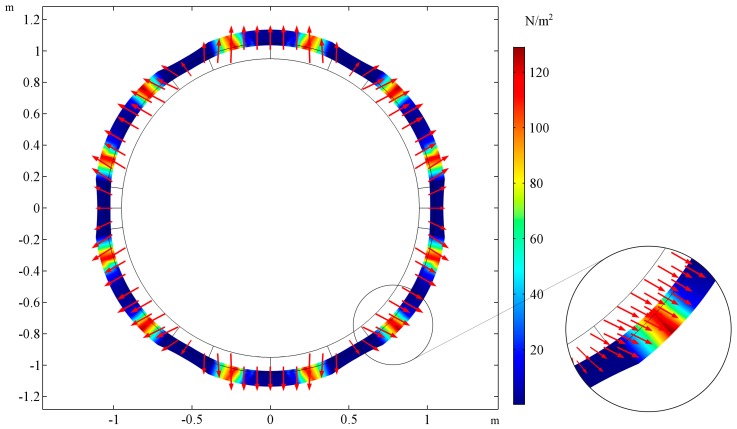
The deformation of the cross-section of the stretched tube and stress field for *E_R_/E_M_* = 1000, the auxeticity of the reinforcement determines the behavior of the tube. The tube’s wall thickness is equal to the reinforcement thickness, inner, and outer layers (L_1_, L_3_) do not occur: *t_h_* = 0. The area of stress concentration is magnified on the right.

The shape of the deformed cross section is presented for two cases. In Figure 3, the cross-sectional shape of the tube is presented for the following set of parameters: *E_R_/E_M_* = 0.001, *ν_R_ = ν_M_ = 0.3*, *t_h_* = 0. The cross-sectional shape for the same parameters but the ratio of Young’s moduli changed *E_R_/E_M_* = 1000 is presented in the Figure 4. The selected cross-section lays in the plane of tube symmetry. The stiffness of the auxetic reinforcement is far smaller than the stiffness of the matrix material. The stresses are concentrated in the area of the matrix in the case of *E_R_/E_M_* = 0.001, while for *E_R_/E_M_* = 1000, the highest values of von Mises stresses occur at the corners of the auxetic structure. Additional red arrows show the directions of displacements so that it is clearly noticeable that the first tube shrinks while the latter expands.

### 3.2. Case 2

**Figure 5:**
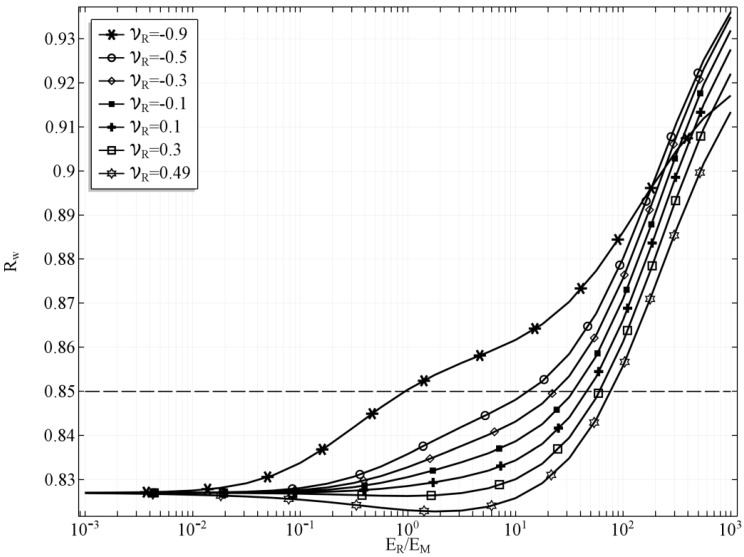
The inner radius of the tube *R_w_* with respect to *E_R_/E_M_* ratio for selected values of *ν_R_*, *t_h_* = 0.2.

In the second analysis, the same auxetic structure is embedded in the matrix material so that *t_h_* = 0.2 and the volumetric share of the reinforcement material $S_{V,R\;}$ = 0.15. The change of the inner radius of the tube *R_w_* with respect to *E_R_/E_M_* ratio for selected values of *ν_R_* is presented in the Figure 5. For such a composite, it is still possible to influence its behaviour by changing the *E_R_/E_M_* ratio, however, significant change is obtained only if *E_R_/E_M_* > 1. Since the share of the reinforcement phase is much smaller than in the first case, the effect of its auxetic structure is suppressed if it is made of softer material i.e., the ratio *E_R_/E_M_* < 1. Hence, the effect of increasing the inner radius *R_w_* if the *E_R_/E_M_* tends to 0.001 is not observed in this case. Furthermore, the influence of the *ν_R_* diminishes with the decrease of *E_R_/E_M_,* and it becomes negligibly small for *E_R_/E_M_* < 0.1. Likewise, the first case of the composite tube, this one is also capable of changing the inner radius by ca. 10% if the *E_R_/E_M_* change by about one order of magnitude (e.g., from 12 to 120). It is worth noting that this behaviour is only slightly influenced by the value of *ν_R_,* although the value of the inner radius depends on it.

**Figure 6:**
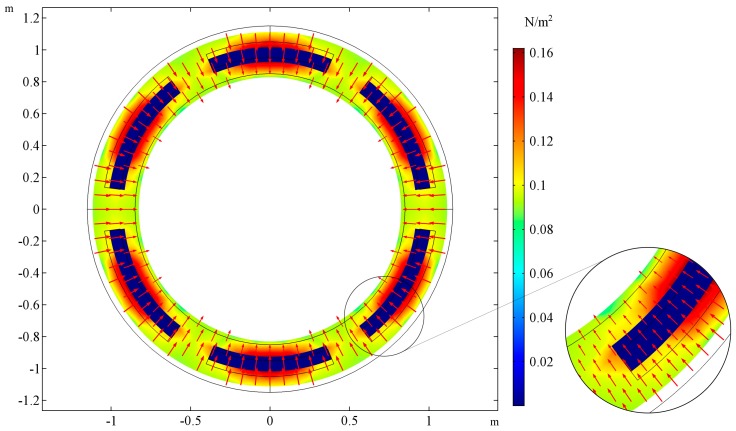
The deformation of the cross-section of the tube stretched and stress field for *E_R_/E_M_* = 0.001, the auxeticity of the reinforcement’s structure is dominated by the behavior of the matrix material. The reinforcement embedded in the matrix material: *t_h_* = 0.2. The area of stress concentration is magnified on the right.

**Figure 7:**
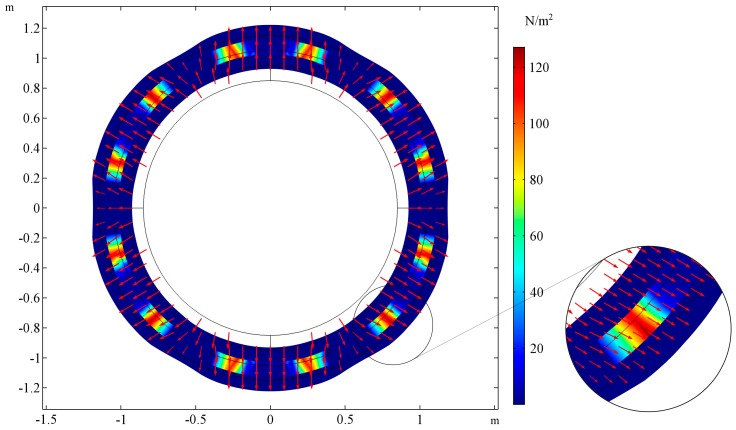
The deformation of the cross-section of the stretched tube and stress field for *E_R_/E_M_* = 1000. The auxeticity of the reinforcement determines the behavior of the tube. The reinforcement embedded in the matrix material: *t_h_* = 0.2. The area of stress concentration is magnified on the right.

The shape of the deformed tube’s cross-section is presented in the Figure 6 in the case of *E_R_/E_M_* = 0.001, whereas Figure 7 shows the cross-sectional shape for *E_R_/E_M_* = 1000 is presented in Figure 7. The highest values of stresses concentrate in the area of the matrix along the interface between phases in the case of *E_R_/E_M_* = 0.001. If the Young’s modulus of the reinforcement is higher than the one of the matrix i.e *E_R_/E_M_* = 1000, then the highest values of the stresses occur at the corners of the auxetic reinforcement, as it occurred for the geometry with *t_h_* = 0.

**Figure 8:**
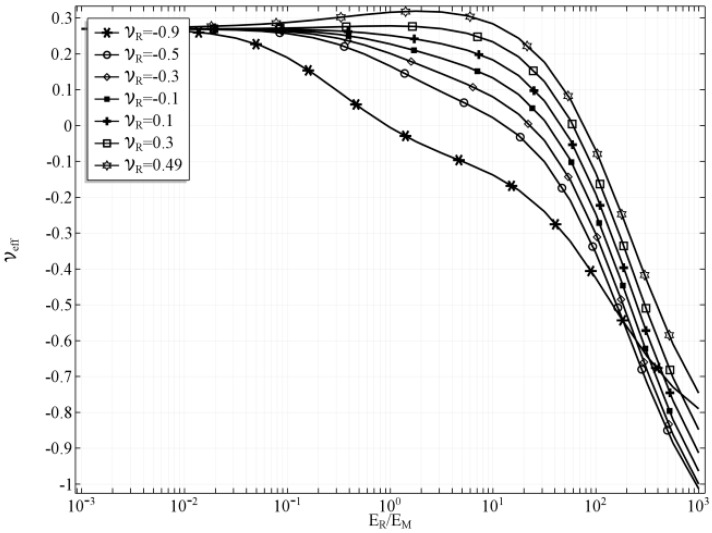
Effecive Poisson’s ratio with respect to *E_R_/E_M_* ratio for selected values of *ν_R_*, *t_h_* = 0.2.

The values of effective Poisson’s ratio of the composite were also calculated (see Figure 8). Since the value of the effective Poisson’s ratio is strongly related to the change of the tube’s radius, the plot in the Figure 8 resembles the plot in the Figure 5. 

## 4. Conclusions

In this paper, a composite tube that is reinforced with auxetic structure is studied by means of Finite Element Analysis. The reinforcement of the tube is assumed to be made of the material whose Young’s modulus can be controlled by external factor e.g., magnetic field, temperature, or other physical phenomena, while the other Young’s modulus is constant. Hence, the ratio of both Young’s moduli changes as well. The change of this ratio allows for controlling the deformation of the composite tube. The shape of reinforcement is designed as auxetic re-entrant structure. Two cases of the tube are considered. In the first case, the tube’s wall is only as thick as the reinforcement layer, and the matrix materials fill the voids of the auxetic structure. The results indicate that the diameter of the stretched tube can change about 10% as a result of the external field applied. In the second analysis, the reinforcement is embedded in the matrix material so the influence of its auxetic geometry is diminished. Yet, the results obtained indicate that it is also possible to control the diameter of such a tube in the range of about 10%, although the changes are limited to the case in which the value of Young’s modulus of the reinforcement is greater than the Young’s modulus of the matrix. Further research will be conducted in order to investigate the behaviour of such structure under different types of loading.

The reinforcement can be completely embedded in the matrix i.e., it is possible to isolate it from the surroundings if necessary.
